# Differential symptom relief profiles of menopausal therapies: an online survey study

**DOI:** 10.1186/s12905-025-03929-3

**Published:** 2025-08-04

**Authors:** Nayra A. Martin-Key, Erin L. Funnell, Jakub Tomasik, Sabine Bahn

**Affiliations:** Cambridge Centre for Neuropsychiatric Research, Department of Chemical Engineering and Biotechnology, University of Cambridge, Philippa Fawcett Drive, Cambridge, CB3 0AS UK

**Keywords:** HRT, Hormone replacement therapy, Women’s health, Menopause, Perimenopause, Post-menopause, Treatment, Testosterone, MENQOL, Symptom relief

## Abstract

**Background:**

While there exist safe hormonal and non-hormonal therapeutic interventions for the menopause, their efficacy profiles are not fully characterized. This study sought to use a symptom checklist to examine menopausal symptom relief associated with different treatments.

**Methods:**

An online survey study was conducted between December 2023 and February 2024. Convenience sampling was conducted, with participants recruited via social media, email, through relevant foundations and support groups, and by word-of-mouth. Inclusion criteria were: (1) ≥ 18 years, (2) assigned female at birth, (3) strong comprehension of the English language, and (4) must be *currently* experiencing symptoms of the menopause or menopause transition. 3330 respondents consented to participate in the study and of these, 91.95% (*N* = 3062) who had completed at least 88% of the survey were included in the analysis. Symptom relief per treatment (transdermal hormone replacement therapy (HRT), oral HRT, vaginal HRT, antidepressants, testosterone, cognitive behavioral therapy (CBT)/other therapy/counseling) was assessed using the symptoms included in the Menopause-Specific Quality of Life (MENQOL) questionnaire, which measures four symptom domains: vasomotor, psychosocial, physical, and sexual.

**Results:**

Data from a total of 3062 respondents were included for analysis (mean age = 51.97, SD = 5.24). Treatment response rates differed significantly across the domains (vasomotor: *F*(5,2340) = 204.93, *p* < 0.001, η^2^ = 0.31; psychosocial: *F*(5,2340) = 75.12, *p* < 0.001, η^2^ = 0.14; physical: *F*(5,2340) = 65.46, *p* < 0.001, η^2^ = 0.12; sexual: *F*(5,2340) = 89.34, *p* < 0.001, η^2^ = 0.16). Transdermal HRT performed better at reducing vasomotor symptoms relative to all other treatment options. Regarding psychosocial symptoms, CBT/other therapy/counseling outperformed all other treatment options. The use of transdermal HRT and testosterone was associated with greater response rates in physical symptoms relative to other treatments. Finally, vaginal HRT and testosterone were associated with significantly higher response rates in sexual symptoms in comparison to all other treatments.

**Conclusions:**

The findings demonstrate differential response rates to menopausal treatments across symptom domains, underscoring the importance of a comprehensive, multidimensional approach to menopausal symptom management. Utilizing a symptom checklist can facilitate the tailoring of treatment plans for specific symptom profiles and patient needs. The outcomes of this study hold considerable implications for improving and shaping treatment guidelines for the menopause.

**Supplementary Information:**

The online version contains supplementary material available at 10.1186/s12905-025-03929-3.

## Background

The menopause is characterized by the permanent halt of menstruation. While it typically occurs naturally between the ages of 44 and 55 years [1] due to declining ovarian follicular activity [2], the menopause can, for instance, be induced earlier through surgical procedures, medication, or severe illness [3]. Women spend approximately one-third of their lifespan in the post-menopause [4] and projections indicate that by 2025, the global count of post-menopausal women will surpass 1 billion [5]. 

Despite its ubiquitousness, the symptoms linked to the menopause and its transition can pose significant challenges. Up to 80% of women experience difficulties during this period, and 25% categorize these challenges as severe [6]. Both the perimenopause and post-menopause phases are commonly linked to vasomotor (e.g., hot flushes and night sweats), physical (e.g., tiredness and bone and joint pain), and sexual symptoms (e.g., loss of libido and vaginal dryness during intercourse) that can profoundly affect an individual’s quality of life [7–12]. Moreover, the menopause, especially during the transitional period, may elevate the risk of mental health issues [13], including depression and anxiety [14, 15] and even suicidal ideation [16]. Certainly, the menopause is a complex phase of life encompassing not just physical changes but also notable psychological challenges. Acknowledging the holistic nature of menopausal experiences is essential for delivering comprehensive care.

Notably, HRT remains the most effective treatment for vasomotor symptoms [17] and may also help alleviate menopause-related depressive symptoms [18, 19], though there is evidence to suggest that these symptoms could get worse after discontinuation [20]. Further, given that menopause is a result of changes in hormonal levels, such supplementation is likely to improve symptoms associated with the menopause as well as have a protective effect on multiple organ systems [21]. 

Antidepressants may be a viable treatment option for mood-related menopause symptoms [22], particularly in those presenting with contraindications for HRT. However, there exists controversy over the frequent use of antidepressants for the reduction of mental health symptoms associated with the menopause [23, 24]. Antidepressants are only recommended in cases where individuals are suspected to have or have a diagnosis of depression, and first line treatment recommendations for menopause related depressive symptoms are HRT and CBT [22]. Despite this, a recent study examining perceptions of healthcare provision for the menopause in the UK demonstrated that women tend to feel dissatisfied with the management of their treatment options for the menopause, often reporting instances where they had perceived that HCPs had made potentially inappropriate recommendations for antidepressants or other psychiatric medication instead of HRT [25]. For those who prefer non-medical treatment choices for psychosocial symptoms of the menopause, cognitive behavioral therapy (CBT) has been deemed a viable option and has been shown to target low mood and anxiety, as well as vasomotor symptoms, and is recommended for hot flushes and night sweats [26, 27]. In regards to sexual symptoms related to the menopause, testosterone has been shown to significantly enhance sexual function and is recommended by the National Institute for Health and Care Excellence (NICE) guidelines for menopausal women with low libido when HRT alone is ineffective [28, 29]. However, testosterone therapy for women remains a controversial topic, largely due to the limited long-term safety data available [30]. 

Taken together, while evidence supports the efficacy of various menopause treatments, findings are inconsistent across studies, and controversy persists over certain options. Critically, significant disparities exist in the global availability and licensing of treatment and support options for the menopause [31]. Additionally, there is a lack of understanding about women’s perceptions of treatment efficacy for specific menopausal symptoms or symptom profiles. This research aimed to address this gap by using a symptom checklist to examine self-reported symptom relief associated with different treatments in a large, multinational sample of menopausal women. The aim of this analysis is not to identify the best therapies to manage or alleviate menopausal symptoms, but to focus on the comparative efficacy of different treatment approaches in reducing specific menopausal symptoms.

## Methods

### Participants

Participants were recruited between December 2023 and February 2024 via email, paid Facebook and Instagram advertisements, free posts on the Cambridge Centre for Neuropsychiatric Research Facebook and X (formerly known as Twitter) websites, and Reddit. Recruitment messages were also disseminated by word-of-mouth and through relevant foundations and support groups.

Inclusion criteria for the study were: (1) ≥ 18 years, (2) assigned female at birth, (3) strong comprehension of the English language, and (4) must be *currently* experiencing symptoms of the menopause or menopause transition (e.g., hot flushes, mood changes, night sweats, irregular or absent periods, decreased sex drive). Participants were also required to not be currently pregnant or breastfeeding or to have given birth in the last year. Participants did not have to have sought help for their menopausal symptoms from a HCP (including not having sought any therapeutic or management advice) or to have used or taken any treatment or support options for the menopause to take part. Participants were not offered reimbursement for their participation in the current study.

### Materials and procedure

An online survey was created using Qualtrics XM^®^. The survey questions and accompanying study materials were designed by a senior research associate (NMK) in consultation with a research assistant (EF) and an experienced psychiatrist (SB). All team members involved in survey design have experience in developing surveys for the menopause [32–34]. The survey could be completed in 15–20 min and was adaptive in nature, such that only relevant questions were asked based on previous responses. For the purpose of the current analysis, only data pertaining to sociodemographic characteristics and symptom relief per treatment option (i.e., transdermal HRT, oral HRT, vaginal HRT, antidepressants, testosterone, and CBT/other type of therapy/counseling) were included.

Menopause status was based on the definitions put forth by the Study of Women’s Health Across the Nation (SWAN) [35], whereby participants were required to select which of the following options best described their experience of the menopause: (a) early perimenopause stage: significant change in the length of menstrual bleed or the time between periods that is not due to pregnancy or breastfeeding, stress or a medical condition; (b) late perimenopause stage: no menstrual bleeding in 3–11 months not due to pregnancy or breastfeeding, stress, or a medical condition, (c) post-menopause: no menstrual bleeding in 12 months not due to pregnancy or breastfeeding, stress or a medical condition, or (d) medically induced menopause: no menstrual bleeding due to hysterectomy (i.e., removal of the uterus) and/or one or two ovaries removed or another medical procedure.

Symptom relief per individual treatment option (current use ≥ 3months, assessed with a question asking how long they had been using each endorsed treatment if currently being used) was assessed by asking participants to select the symptoms that had improved (“*Which of the following menopause symptoms has your current [treatment] helped improve? Select all that apply to you.”*) for each treatment option currently being used by ticking a box (ticked = yes, not ticked = no). Symptoms were presented as a list and consisted of the 29 symptoms included in the Menopause-Specific Quality of Life (MENQOL) [36] questionnaire, which measures four symptom domains: vasomotor, psychosocial, physical, and sexual. This approach was favored over asking the MENQOL questionnaire each time to reduce participant burden by reducing the survey administration time.

### Data analytic strategy

Data were processed and analyzed in SPSS version 28.0.1.1. and R 4.3.0. Symptom domain relief scores were calculated by summing the presence of improvements (yes/no) in symptoms pertaining to each domain. Scores were scaled between 0 and 1 for comparability across the four domains. We scaled the cumulative symptom domain scores to the range 0–1 to allow for comparability across domains, as each domain in the MENQOL contains a different number of symptoms. Using raw counts would have introduced bias, as domains with more items could show greater absolute change due to their higher symptom count. In turn, scaling enabled a direct comparison of relative symptom relief across domains.

While each symptom was scored categorically (yes/no for relief), the aggregated and scaled domain-level scores reflect the proportion of symptoms within that domain that improved, yielding a quasi-continuous variable suitable for analysis using ANCOVA. This is a common approach when working with summed outcomes [37, 38]. It is also statistically equivalent to the standard MENQOL scoring method, which involves calculating mean scores across items within each domain [39, 40]. 

Differences in symptom domain relief scores by treatment option (current use ≥ 3months) were analyzed using one-way ANCOVAs, with effect sizes reported as eta-squared (η^2^; small = 0.01, medium = 0.06, large = 0.14) [41]. The analysis was adjusted for age, gender, country of residence, menopause status (perimenopause/post-menopause/medically-induced menopause), ethnicity, education, employment, and concurrent use of menopausal treatments (yes/no; number). Pairwise post-hoc comparisons between treatments were adjusted for multiple comparisons using the Bonferroni correction.

To account for the potential use of hormonal treatments in respondents that respondents who did not identify as women may have been taking or using concurrently, analyses were repeated excluding those who identified as men, non-binary, and other, and well as those who selected ‘prefer not to answer’.

### Ethical approval and informed consent

The study was approved by the University of Cambridge Psychology Research Ethics Committee (approval number PRE.2023.123). All participants provided informed consent electronically to participate in the study. All study procedures were carried out inline with principles as set out in the Declaration of Helsinki.

## Results

### Overview

3330 respondents consented to participants in the study. 91.95% (*N* = 3062) who had completed at least 88% of the survey were included in the analysis.

Respondents’ sociodemographic and treatment characteristics are shown in Table 1. The mean age was 51.97 (*SD* = 5.24), with the majority of respondents identifying with the female gender (98.76%, *n* = 3024), being white (90.01%, *n* = 2756), having at least an undergraduate degree (69.17%, *n* = 2118), and being in current employment (i.e., full-time, part-time, self-employed; 80.96%, *n* = 2479).

**Table 1:** Sociodemographic and treatment characteristics (*N* = 3062)

	M (SD)	
Age		51.97 (5.24)
	***n*** **(%)**	
Gender	Woman	3024 (98.76)
	Man	5 (0.16)
	Non-binary	20 (0.65)
	Other	3 (0.10)
	Prefer not to answer	10 (0.33)
Country of residence	Australia	508 (16.59)
	Canada	537 (17.54)
	New Zealand	508 (16.59)
	United Kingdom	735 (24.00)
	United States of America	774 (25.28)
Menopause status	Perimenopause	1387 (45.30)
	Post-menopause	1218 (39.78)
	Medically-induced menopause	457 (14.92)
Ethnicity	White/Caucasian	2756 (90.01)
	Asian	48 (1.57)
	Black/Caribbean/African	22 (0.72)
	Arab/Middle Eastern/North African descent	7 (0.23)
	Hispanic/Latinx	43 (1.40)
	Mixed/multiple	133 (4.34)
	Other	37 (1.21)
	I am not sure	2 (0.07)
	Prefer not to answer	14 (0.46)
Education	≤ Primary (up to 11 years)	53 (1.73)
	Lower secondary (up to 16 years)	240 (7.84)
	Upper secondary (up to 18 years)	448 (14.63)
	Undergraduate/college degree (i.e., Bachelors)	1207 (39.42)
	Postgraduate degree (e.g., Masters, PhD)	911 (29.75)
	Other	146 (4.77)
	Prefer not to answer	57 (1.86)
Employment ^a^	Full-time	1536 (49.84)
	Part-time	574 (18.75)
	Self-employed	369 (12.05)
	Parental leave/caring responsibilities	46 (1.50)
	Homemaker	268 (8.75)
	Student	40 (1.31)
	Voluntary work	86 (2.81)
	Retired	214 (6.99)
	Unemployed	112 (3.66)
	Prefer not to answer	70 (2.29)
Seen HCP for menopause symptoms	Yes	2485 (81.16)
	No	577 (18.84)
Been prescribed treatment/support for the menopause ^b^	Yes	1906 (76.70)
	No	579 (23.30)
Transdermal HRT ^c^	Yes, currently using	1025 (57.26)
	Yes, in the past	222 (12.40)
	No, never	543 (30.34)
Oral HRT ^c^	Yes, currently using	564 (31.51)
	Yes, in the past	285 (15.92)
	No, never	941 (52.57)
Vaginal HRT ^c^	Yes, currently using	441 (24.64)
	Yes, in the past	205 (11.45)
	No, never	1144 (63.91)
Antidepressants ^c^	Yes, currently using	432 (24.13)
	Yes, in the past	294 (16.42)
	No, never	1064 (59.44)
Testosterone ^c^	Yes, currently using	226 (12.63)
	Yes, in the past	94 (5.25)
	No, never	1470 (82.12)
CBT/other therapy/counseling ^c^	Yes, currently using	210 (11.73)
	Yes, in the past	354 (19.78)
	No, never	1226 (68.49)
Length of current transdermal HRT use ^d^	Less than 3 months	154 (15.02)
	3–6 months	140 (13.66)
	6 months– 1 year	216 (21.07)
	1–2 years	284 (27.71)
	More than 2 years	231 (22.54)
Length of current oral HRT use ^e^	Less than 3 months	92 (16.37)
	3–6 months	80 (14.24)
	6 months– 1 year	106 (18.86)
	1–2 years	142 (25.27)
	More than 2 years	142 (25.27)
Length of current vaginal HRT use ^f^	Less than 3 months	94 (21.32)
	3–6 months	66 (14.97)
	6 months– 1 year	91 (20.64)
	1–2 years	90 (20.41)
	More than 2 years	100 (22.68)
Length of current antidepressant use ^g^	Less than 3 months	76 (17.59)
	3–6 months	31 (7.18)
	6 months– 1 year	66 (15.28)
	1–2 years	74 (17.13)
	More than 2 years	185 (42.82)
Length of current testosterone use ^h^	Less than 3 months	46 (20.35)
	3–6 months	41 (18.14)
	6 months– 1 year	49 (21.68)
	1–2 years	44 (19.47)
	More than 2 years	46 (20.35)
Length of current CBT/other therapy/counseling use ^i^	Less than 3 months	59 (28.64)
	3–6 months	31 (15.05)
	6 months– 1 year	31 (15.05)
	1–2 years	32 (15.53)
	More than 2 years	53 (25.73)

Note. CBT, cognitive behavioral therapy; HCP, health care professional; HRT, hormone replacement therapy

*Key.* ^a^ Percentages add to more than 100% as participants could select multiple options; ^b^ includes those who had visited a HCP for their menopausal symptoms (*n* = 2485); ^c^ includes those who had been prescribed treatment/support for the menopause (*n* = 1790; 116 respondents selected ‘other’ treatment and were not included); ^d^ includes those who were currently using transdermal HRT (*n* = 1025); ^e^ includes those who were currently taking oral HRT (*n* = 562; missing data from two respondents as they had initially selected ‘past use’ but open-ended responses suggested ongoing use); ^f^ includes those who were currently using vaginal HRT (*n* = 441); ^g^ includes those who were currently taking antidepressants (*n* = 432); ^h^ includes those who were currently using testosterone (*n* = 226); ^i^ includes those who were currently using CBT/other therapy/counseling (*n* = 206; missing data from four respondents as they had initially selected ‘past use’ but open-ended responses suggested ongoing use)

81.16% (*n* = 2485) of participants had discussed their menopausal symptoms with a HCP. Of these, 76.70% (*n* = 1906) had been prescribed treatment, with the most common treatment options (i.e., current or past use) being transdermal HRT (69.66%, *n* = 1247), oral HRT (47.43%, *n* = 849), and antidepressants (40.46%, *n* = 726). For an overview of respondents’ concurrent treatment use see Supplementary Fig. 1 (Supplementary Information).

**Fig. 1:**
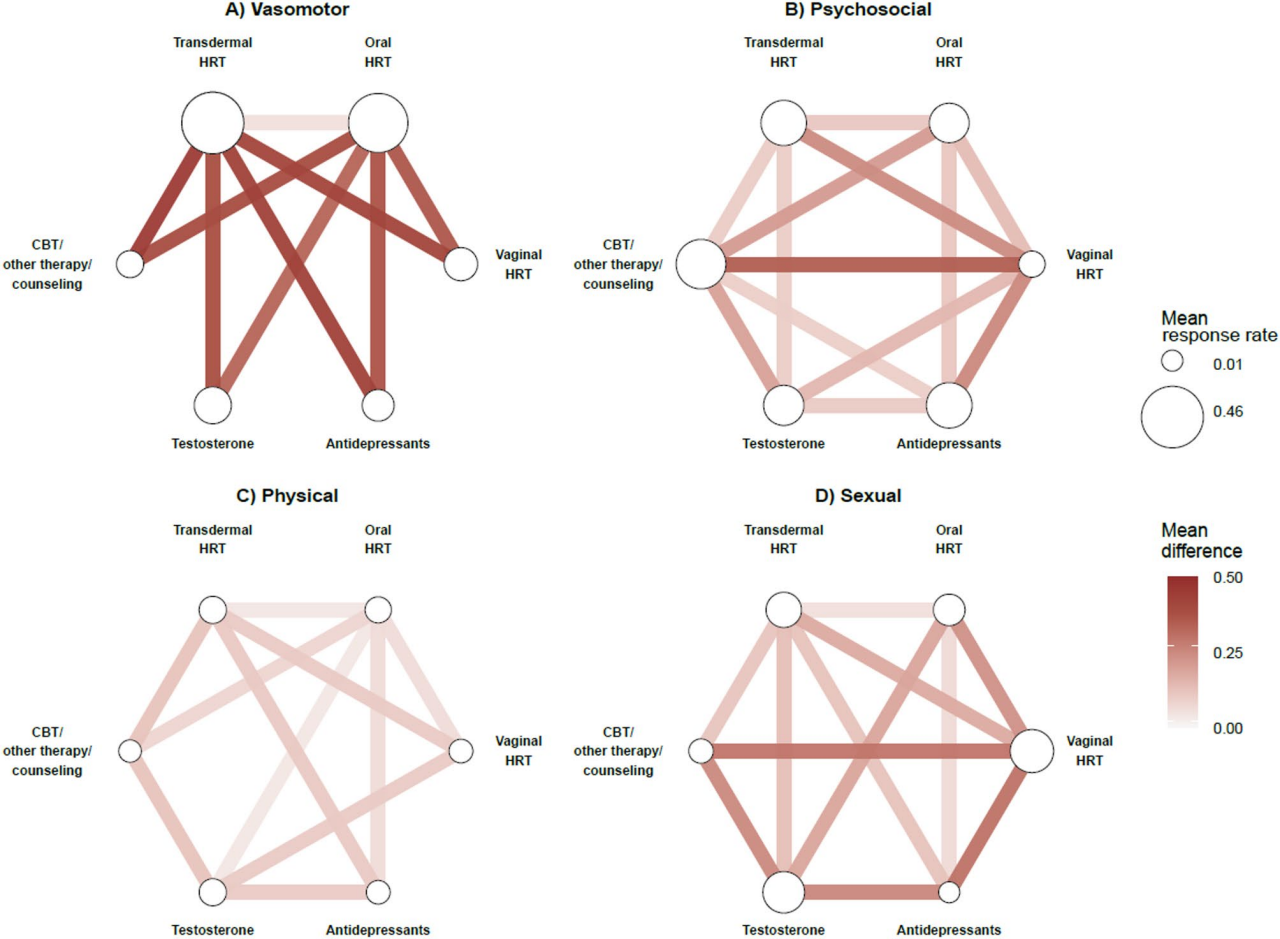
Differential responses to treatment across the MENQOL symptom domains. Shown are pairwise comparisons of treatment options per domain: (**A**) vasomotor, (**B**) psychosocial, (**C**) physical, and (**D**) sexual. The size of the circles corresponds to the mean response rate across symptoms within a domain, scaled to 0–1 and adjusted for the covariates. Lines represent significant (Bonferroni-adjusted) differences in response rates between treatments. *Key.* CBT, cognitive behavioral therapy; HRT, hormone replacement therapy; MENQOL, Menopause-Specific Quality of Life Questionnaire

### Symptom relief

An overview of menopausal symptom relief per treatment option is shown in Fig. 1 and Supplementary Table 1 (Supplementary Information). Response rates (the % of participants who reported responding to the treatment) for the treatment options differed significantly across the domains (vasomotor: *F*(5,2340) = 204.93, *p* < 0.001, η^2^ = 0.31; psychosocial: *F*(5,2340) = 75.12, *p* < 0.001, η^2^ = 0.14; physical: *F*(5,2340) = 65.46, *p* < 0.001, η^2^ = 0.12; sexual: *F*(5,2340) = 89.34, *p* < 0.001, η^2^ = 0.16).

**Fig. 2:**
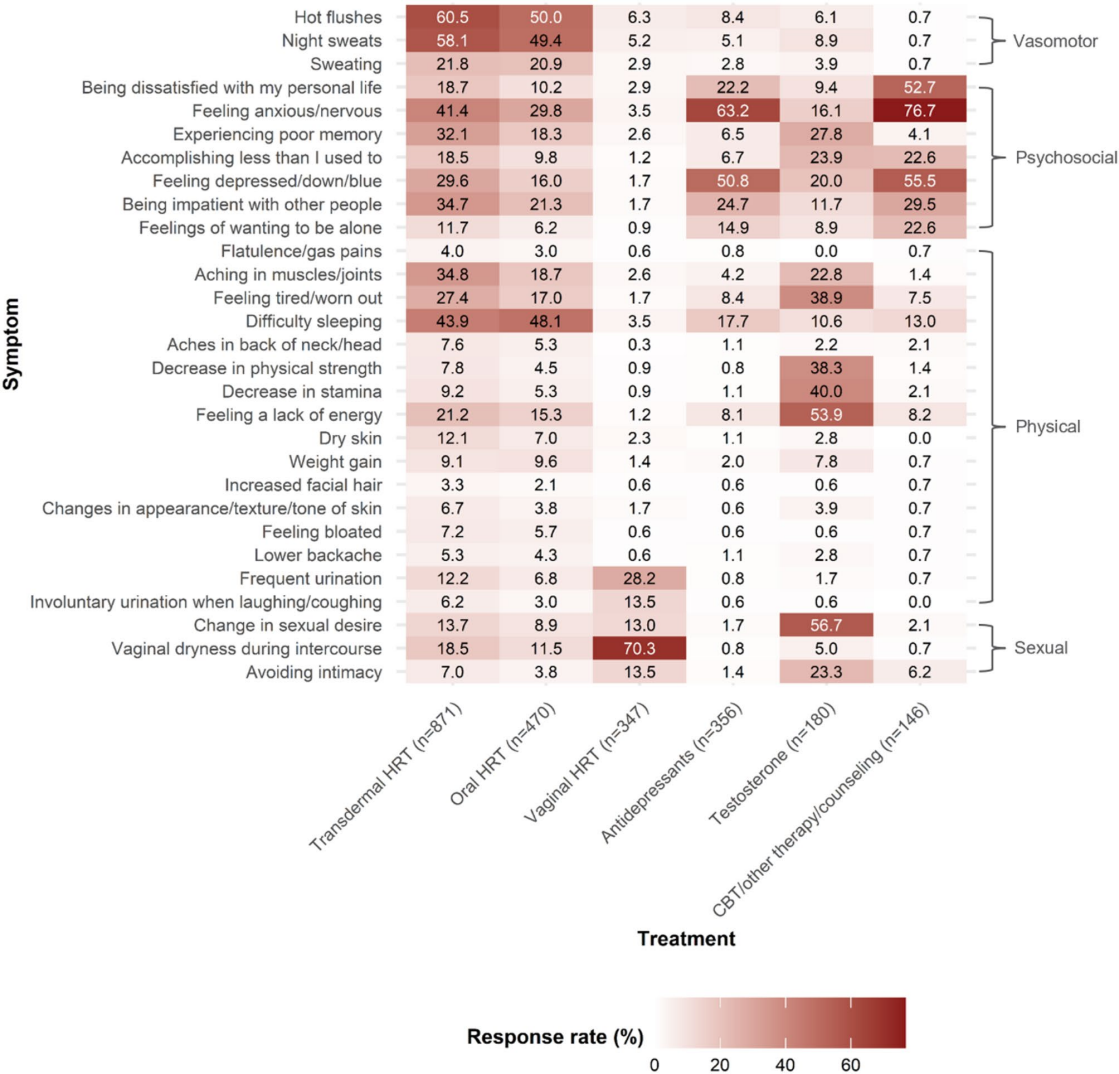
Treatment response (% responders) for the 29 menopausal symptoms from the Menopause-Specific Quality of Life Questionnaire (MENQOL; grouped by domain) per treatment option. *Key.* CBT, cognitive behavioral therapy; HRT, hormone replacement therapy

For an overview of Bonferroni-corrected pairwise post-hoc comparisons see Fig. 2; Tables 2 and 3. Regarding vasomotor symptoms, the use of transdermal HRT was associated with greater response rates relative to all other treatment options (all *p* ≤ 0.02), while the use of oral HRT was related with higher response rates in comparison to vaginal HRT, antidepressants, testosterone, and CBT/other therapy/counseling (all *p* ≤ 0.001).

**Table 2:** Mean response rate (the % of participants who reported responding to the treatment) per treatment for each MENQOL symptom domain (current use ≥ 3months)

MENQOL symptom domain	Treatment	Mean response rate ^a^	95% CI
Vasomotor	Transdermal HRT	0.46	0.44–0.48
	Oral HRT	0.41	0.38–0.43
	Vaginal HRT	0.05	0.02–0.08
	Antidepressants	0.04	0.01–0.07
	Testosterone	0.08	0.04–0.12
	CBT/other therapy/counseling	0.02	-0.03–0.07
Psychosocial	Transdermal HRT	0.27	0.25–0.28
	Oral HRT	0.16	0.14–0.18
	Vaginal HRT	0.02	0.00–0.05
	Antidepressants	0.27	0.25–0.30
	Testosterone	0.17	0.13–0.20
	CBT/other therapy/counseling	0.37	0.33–0.41
Physical	Transdermal HRT	0.14	0.13–0.15
	Oral HRT	0.10	0.09–0.11
	Vaginal HRT	0.04	0.02–0.05
	Antidepressants	0.03	0.02–0.05
	Testosterone	0.14	0.12–0.16
	CBT/other therapy/counseling	0.02	0.00–0.04
Sexual	Transdermal HRT	0.14	0.12–0.15
	Oral HRT	0.08	0.06–0.10
	Vaginal HRT	0.31	0.29–0.34
	Antidepressants	0.01	-0.01–0.04
	Testosterone	0.27	0.24–0.30
	CBT/other therapy/counseling	0.02	-0.02–0.06

Note. CBT, cognitive behavioral therapy; CI, confidence interval; HRT, hormone replacement therapy

*Key.* ^a^ Scaled to 0–1 and adjusted for the covariates (age, gender, country of residence, menopause status (perimenopause/post-menopause/medically-induced menopause), ethnicity, education, employment, and concurrent use of menopausal treatment (yes/no; number)

**Table 3:** Mean differences in response rates between treatments per MENQOL symptom domain (current use ≥ 3months)

MENQOL symptom domain	Treatment comparisons ^a^		Mean difference ^b^	95% CI	*p*
Vasomotor	Transdermal HRT	Oral HRT	0.05	0.00–0.10	0.023*
	Transdermal HRT	Vaginal HRT	0.41	0.35–0.47	< 0.001***
	Transdermal HRT	Antidepressants	0.42	0.36–0.47	< 0.001***
	Transdermal HRT	Testosterone	0.38	0.31–0.46	< 0.001***
	Transdermal HRT	CBT/other therapy/counseling	0.44	0.36–0.52	< 0.001***
	Oral HRT	Vaginal HRT	0.36	0.30–0.42	< 0.001***
	Oral HRT	Antidepressants	0.37	0.30–0.43	< 0.001***
	Oral HRT	Testosterone	0.33	0.25–0.41	< 0.001***
	Oral HRT	CBT/other therapy/counseling	0.39	0.30–0.47	< 0.001***
	Vaginal HRT	Antidepressants	0.01	-0.06–0.08	1.000
	Vaginal HRT	Testosterone	-0.03	-0.11–0.05	1.000
	Vaginal HRT	CBT/other therapy/counseling	0.03	-0.06–0.11	1.000
	Antidepressants	Testosterone	-0.04	-0.12–0.04	1.000
	Antidepressants	CBT/other therapy/counseling	0.02	-0.07–0.11	1.000
	Testosterone	CBT/other therapy/counseling	0.06	-0.04–0.15	1.000
Psychosocial	Transdermal HRT	Oral HRT	0.11	0.07–0.15	< 0.001***
	Transdermal HRT	Vaginal HRT	0.24	0.20–0.29	< 0.001***
	Transdermal HRT	Antidepressants	0.00	-0.05–0.04	1.000
	Transdermal HRT	Testosterone	0.10	0.04–0.16	< 0.001***
	Transdermal HRT	CBT/other therapy/counseling	-0.10	-0.16– -0.04	< 0.001***
	Oral HRT	Vaginal HRT	0.14	0.09–0.18	< 0.001***
	Oral HRT	Antidepressants	-0.11	-0.16– -0.06	< 0.001***
	Oral HRT	Testosterone	-0.01	-0.07–0.05	< 0.001***
	Oral HRT	CBT/other therapy/counseling	-0.21	-0.27– -0.14	< 0.001***
	Vaginal HRT	Antidepressants	-0.25	-0.30– -0.20	< 0.001***
	Vaginal HRT	Testosterone	-0.15	-0.21– -0.08	< 0.001***
	Vaginal HRT	CBT/other therapy/counseling	-0.34	-0.41– -0.27	< 0.001***
	Antidepressants	Testosterone	0.10	0.04–0.17	< 0.001***
	Antidepressants	CBT/other therapy/counseling	-0.10	-0.16– -0.03	0.001**
	Testosterone	CBT/other therapy/counseling	-0.20	-0.27– -0.12	< 0.001***
Physical	Transdermal HRT	Oral HRT	0.04	0.02–0.06	< 0.001***
	Transdermal HRT	Vaginal HRT	0.10	0.08–0.13	< 0.001***
	Transdermal HRT	Antidepressants	0.11	0.08–0.13	< 0.001***
	Transdermal HRT	Testosterone	0.00	-0.03–0.03	1.000
	Transdermal HRT	CBT/other therapy/counseling	0.12	0.09–0.16	< 0.001***
	Oral HRT	Vaginal HRT	0.06	0.04–0.09	< 0.001***
	Oral HRT	Antidepressants	0.07	0.04–0.09	< 0.001***
	Oral HRT	Testosterone	-0.04	-0.07– -0.01	0.006**
	Oral HRT	CBT/other therapy/counseling	0.08	0.04–0.12	< 0.001***
	Vaginal HRT	Antidepressants	0.00	-0.03–0.03	1.000
	Vaginal HRT	Testosterone	-0.10	-0.14– -0.07	< 0.001***
	Vaginal HRT	CBT/other therapy/counseling	0.02	-0.02–0.05	1.000
	Antidepressants	Testosterone	-0.11	-0.14– -0.07	< 0.001***
	Antidepressants	CBT/other therapy/counseling	0.02	-0.02–0.05	1.000
	Testosterone	CBT/other therapy/counseling	0.12	0.08–0.16	< 0.001***
Sexual	Transdermal HRT	Oral HRT	0.06	0.02–0.09	< 0.001***
	Transdermal HRT	Vaginal HRT	-0.18	-0.22– -0.13	< 0.001***
	Transdermal HRT	Antidepressants	0.12	0.08–0.16	< 0.001***
	Transdermal HRT	Testosterone	-0.13	-0.19– -0.08	< 0.001***
	Transdermal HRT	CBT/other therapy/counseling	0.12	0.06–0.18	< 0.001***
	Oral HRT	Vaginal HRT	-0.23	-0.28– -0.19	< 0.001***
	Oral HRT	Antidepressants	0.07	0.02–0.11	< 0.001***
	Oral HRT	Testosterone	-0.19	-0.24– -0.13	< 0.001***
	Oral HRT	CBT/other therapy/counseling	0.06	0.00–0.12	0.053
	Vaginal HRT	Antidepressants	0.30	0.25–0.35	< 0.001***
	Vaginal HRT	Testosterone	0.04	-0.02–0.10	0.459
	Vaginal HRT	CBT/other therapy/counseling	0.29	0.23–0.36	< 0.001***
	Antidepressants	Testosterone	-0.26	-0.32– -0.19	< 0.001***
	Antidepressants	CBT/other therapy/counseling	-0.01	-0.07–0.06	1.000
	Testosterone	CBT/other therapy/counseling	0.25	0.18–0.32	< 0.001***

Note. CBT, cognitive behavioral therapy; CI, confidence interval; HRT, hormone replacement

*Key.* ^a^ Post-hoc pairwise comparisons, subject to the Bonferroni-correction method; ^b^ Scaled to 0–1 and adjusted for the covariates (age, gender, country of residence, menopause status (perimenopause/post-menopause/medically-induced menopause), ethnicity, education, employment, and concurrent use of menopausal treatment (yes/no; number); * *p* < 0.05; ** *p* < 0.01; *** *p* < 0.001

In terms of psychosocial symptoms, CBT/other therapy/counseling outperformed all other treatment options (all *p* ≤ 0.001), while both transdermal HRT and antidepressant use were associated with significantly higher response rates in comparison to oral HRT, vaginal HRT, and testosterone (all *p* < 0.001). Oral HRT use was seen to be related with greater response rates for psychosocial symptoms than vaginal HRT (*p* < 0.001).

Testosterone medication was associated with significantly greater response rates for physical symptoms relative to oral HRT, vaginal HRT, antidepressants, and CBT/other therapy/counseling (all *p* ≤ 0.01). The use of transdermal and oral HRT were related to significantly higher response rates for physical symptoms in comparison to vaginal HRT, antidepressants, and CBT/other therapy/counseling (all *p* < 0.001), while transdermal HRT was further associated with better response rates for physical symptoms relative to oral HRT (*p* < 0.001).

Finally, in terms of sexual symptoms, vaginal HRT and testosterone were both independently associated with significantly higher response rates in comparison to all other treatment options (all *p* < 0.001). Both transdermal and oral HRT significantly outperformed antidepressants, demonstrating greater response rates for sexual symptoms (all *p* < 0.001), while transdermal HRT use was also associated with significantly greater response rates than oral HRT for sexual symptoms (*p* < 0.001).

In order to account for the potential use of hormonal treatments that respondents who did *not* identify as women may have been taking or using concurrently, analyses were repeated excluding those who identified as men, non-binary, and other, as well as those who selected ‘prefer not to answer’. All findings from the original analysis remained significant.

## Discussion

The current study aimed to examine self-reported menopausal symptom relief across various treatment options using a symptom checklist in a large multinational sample of menopausal women. The findings from this research highlight differential response rates to menopausal treatments across various symptom domains, underscoring the necessity of a comprehensive, multidimensional approach to menopausal symptom management. This reflects the NICE recommendations for menopause which also highlight the importance of individualized care which is responsive to changes in the symptom profile [22]. Overall, the outcomes of this study hold considerable implications for improving and shaping treatment guidelines for menopause, promoting a more individualized and effective approach to care that enhances the quality of life for menopausal women worldwide.

Previous research indicates that women frequently view their doctors as overly cautious in prescribing hormone-based treatments such as hormone replacement therapy (HRT) [25, 42, 43], with this likely due to nearly two decades of pervasive, conflicting, and frequently alarming information about menopause treatment reaching HCPs and the wider public. Despite these concerns, HCPs may want to prioritize the use of transdermal HRT due to its broad symptom response rates across domains as observed in the current study. Additionally integrating psychological therapies into menopause management would confer a more holistic approach, addressing vasomotor and psychological symptoms. In fact, CBT may have an effect on vasomotor symptoms with some studies having reported reductions in the subjective frequency of hot flashes following CBT [44–48]. However, the current study indicated minimal impact of psychological therapies on vasomotor symptoms. While transdermal HRT and antidepressant use demonstrated similar response rates for psychosocial symptoms, psychological therapies appeared to be associated with even greater response rates. These therapies not only help address psychosocial symptoms but also empower women to develop new coping strategies and adopt more positive thinking styles, offering benefits beyond those provided by purely biological treatments, both hormonal and non-hormonal [49, 50]. However, despite the potential benefits suggested by the current study, psychological therapies may not be suitable as a broad recommendation to manage psychosocial symptoms in menopausal women due to the complex factors underlying treatment efficacy, many of which were not considered in the study. Such factors may include openness to psychological therapies, mental health history, and prior experiences with psychological treatment with these factors being implicated in the level of benefits derived for different individuals. Therefore, while psychological therapies were effective for reducing psychosocial symptoms for those who received such treatment in the current study, further work is needed to identify the profiles of individuals who would be most likely to benefit.

Critically, in the current study, the use of psychological therapies was relatively low. To improve accessibility, alternative delivery methods such as online platforms or hybrid models that combine both in-person and online sessions could be explored. Research has shown that patients appreciate the independence and empowerment provided by online platforms [51]. Online applications offer the convenience of access from anywhere with an internet connection, which is particularly beneficial for individuals in remote locations or those with limited mobility [52]. Additionally, online psychological therapies allow women to engage in therapy on their own schedules, making it easier for those with busy lifestyles or difficulties attending traditional sessions regularly [53]. Despite online delivery potentially widening access, barriers may persist in terms of concerns regarding costs and a preference for traditional face-to-face offerings [54] and so it is important to not only tailor the intervention type but also intervention delivery based on individual needs and preferences.

The effectiveness of testosterone and vaginal HRT in addressing sexual dysfunction suggests that these treatments could be integral components of care plans for menopausal women experiencing such symptoms. Notably, testosterone appears effective in alleviating both physical and sexual symptoms, making it a valuable option for a comprehensive treatment strategy. Currently there is mixed evidence regarding the benefits associated with testosterone supplementation. Some studies indicate a general improvement in distressing sexual symptoms [30] and other studies indicate that testosterone is only demonstrably effective for women with hypoactive sexual desire disorder [55]. Unfortunately, the current study did not assess hypoactive sexual desire disorder and therefore it is unclear whether the self-reported improvements in sexual symptoms associated with testosterone use as observed in the current study are specifically related to this condition. Despite this mixed evidence, testosterone is recognized by NICE as a suitable second line offering for menopause related sexual symptoms if HRT has been ineffective. However, testosterone remains poorly addressed in the NICE guidelines. Specifically, the guidelines lack detailed information on prescribing testosterone, appropriate products and dosages, and monitoring requirements [26] and there is a lack of long-term safety data for use of testosterone for relief of menopausal symptoms [56]. Additionally, most countries only offer formulations designed for men, which can result in women being exposed to supraphysiological blood levels and an increased risk of adverse events [30]. Evidently, more research is needed in order to fully understand the effects of testosterone therapy in menopausal women and its long-term safety profile and to improve the confidence of clinical guidance.

Taken together, expanding treatment plans to include a variety of options may ensure that menopausal women receive tailored care that effectively addresses their specific symptom profiles and preferences. Comprehensive assessment of menopausal symptoms is required to facilitate this; however, medical education does not recognize the highly variable nature of menopause symptoms [57] and GPs may be unaware of symptoms other than vasomotor symptoms [58]. This may result in an inflexible view of menopause which has the potential to negatively impact interactions with menopausal patients seeking assessment and support [57]. Utilizing a symptom checklist can further enhance the customization of treatment plans by supporting HCPs in accurately identifying and addressing the unique combination of symptoms experienced by each patient. Moreover, completing these checklists prior to appointments could save valuable clinician time, allowing HCPs to focus more on discussing and implementing treatment strategies, as well as promoting shared decision-making, rather than the identification of troublesome symptoms. Studies indicate a willingness to use symptom checklists by general practitioners (i.e., HCPs in primary care settings where the bulk of menopause care is delivered) but lack awareness of the tools which are available [59]. However, to our knowledge no work has been done to understand how and to what extent such checklists would support HCPs in delivery of menopause care. Future work may also wish to investigate the preferences of patients and HCPs regarding symptom checklists to support their wider deployment in clinical settings.

Notably, pre-consultation interventions, such as checklists, have been shown to increase patient satisfaction in healthcare settings [60]. For instance, checklists provide a pragmatic method for identifying issues in post-stroke patients [61], facilitating referrals to appropriate support services and structuring stroke reviews using a patient-centered approach. Similarly, symptom checklists have been effective in helping clinicians make diagnostic and treatment decisions for primary care patients who report daily cannabis and/or other drug use [62]. By systematically evaluating and tracking symptoms, clinicians can adjust treatments in real-time, ensuring optimal relief and improved patient outcomes. This personalized method not only fosters a more patient-centered approach but is also likely to result in higher levels of satisfaction and adherence to treatment plans. By incorporating symptom checklists and a variety of treatment options, HCPs can deliver more comprehensive and responsive care, ultimately enhancing the quality of life for menopausal women.

### Limitations

Whilst the current survey was developed by a team with experience of delivering survey studies about the menopause, no patient or other relevant HCPs (e.g., GPs, gynecologists, endocrinologists) were consulted. This may have resulted in content oversights and exclusion of therapeutics for menopause that may be regularly prescribed or particularly effective for certain symptoms.

The experiences captured in this study may not fully represent the broader populations of the surveyed countries, including ethnic minorities and disadvantaged groups [63]. Additionally, while social media recruitment and online survey delivery were employed to maximize the sample size, this approach likely introduced recruitment bias. For example, individuals with negative experiences of menopausal treatment may have been more inclined to participate.

Furthermore, the symptom relief data were self-reported and retrospective, potentially introducing biases and inaccuracies. For instance, high levels of concurrent use of transdermal and oral HRT were reported by participants which is clinically unlikely. Additionally, it is worth mentioning that all but one of the symptoms in the MENQOL are negatively worded or presented as unwanted symptoms. It remains unclear whether respondents interpret the remaining item: “a change in sexual desire”, as a negative or positive symptom. Further, because the MENQOL scale was not utilized in this study, it was not possible to quantify the degree to which participants considered symptoms bothersome. As a result, the relative burden of individual menopausal symptoms could not be determined, limiting the ability to draw detailed conclusions about the effect of each treatment on symptom severity.

Finally, duration of use and adherence to intervention were not controlled for in the current study. Different treatments (e.g., psychological therapies vs. HRT) may have different timelines for effectiveness, which could influence outcomes and complicate direct comparisons. These factors should be considered when interpreting the findings.

## Conclusions

This study investigated how well different treatments relieve menopausal symptoms across women from multiple countries using a symptom checklist while adjusting for the relevant covariates, with the overall findings highlighting the need for a comprehensive approach to managing menopause symptoms. HCPs should prioritize the use of transdermal HRT not only for hormonal supplementation of estrogen to reduce harms associated with this deficiency, but because it also improves menopausal symptoms, upgrading, in total, health-related quality of life. Further, incorporating psychological therapies and vaginal HRT or testosterone as needed for potential psychosocial and sexual symptoms, respectively will achieve more holistic care based on symptom presentation. Finally, using a menopausal symptom checklist has the potential to facilitate the customization of treatment.

## Supplementary Information

Below is the link to the electronic supplementary material.

Supplementary Material 1

## Data Availability

The datasets used and/or analysed during the current study are available from the corresponding author on reasonable request.

## Abbreviations

CBT: Cognitive behavioral therapy
HCP: Healthcare professional
HRT: Hormone replacement therapy
MENQOL: Menopause-Specific Quality of Life questionnaire
NICE: National Institute for Health and Care Excellence
SWAN: Study of Women’s Health Across the Nation

## References

[1] Hardy C, Hunter MS, Griffiths A. Menopause and work: an overview of UK guidance. Occup Med. 2018;68(9):580–6. 10.1093/occmed/kqy134 30544239

[2] World Health Organization. World Health Report 2008: Women, Ageing and Health: A Framework for Action. 2008. Available from: https://apps.who.int/iris/bitstream/handle/10665/43810/9789241563529_eng.pdf [Accessed 24 March 2024].

[3] Hoga L, Rodolpho J, Gonçalves B, Quirino B. Womenʼs experience of menopause: a systematic review of qualitative evidence. JBI Database Syst Reviews Implement Rep. 2015;13(8):250–337. 10.11124/jbisrir-2015-1948 26455946

[4] Soules MR, Sherman S, Parrott E, Rebar R, Santoro N, Utian W, et al. Executive summary: stages of reproductive aging workshop (STRAW). Fertil Steril. 2001;76(5):874–8. 11704104 10.1016/s0015-0282(01)02909-0

[5] Shifren JL, Gass MLS. The North American menopause society recommendations for clinical care of midlife women. Menopause. 2014;21(10):1038–62. 25225714 10.1097/GME.0000000000000319

[6] Avis NE, Crawford SL, Greendale G, Bromberger JT, Everson-Rose SA, Gold EB, et al. Duration of menopausal vasomotor symptoms over the menopause transition. JAMA Intern Med. 2015;175(4):531. 25686030 10.1001/jamainternmed.2014.8063 PMC4433164

[7] Dave FG, Adedipe T, Disu S, Laiyemo R. Unscheduled bleeding with hormone replacement therapy. Obstetrician Gynaecologist. 2019;21(2):95–101.

[8] Karaçam Z, Şeker SE. Factors associated with menopausal symptoms and their relationship with the quality of life among Turkish women. Maturitas. 2007;58(1):75–82. 17681681 10.1016/j.maturitas.2007.06.004

[9] Avis NE, Ory M, Matthews KA, Schocken M, Bromberger J, Colvin A. Health-Related quality of life in a multiethnic sample of Middle-Aged women. Med Care. 2003;41(11):1262–76. 14583689 10.1097/01.MLR.0000093479.39115.AF

[10] Avis NE, Colvin A, Bromberger JT, Hess R, Matthews KA, Ory M, et al. Change in health-related quality of life over the menopausal transition in a multiethnic cohort of middle-aged women. Menopause. 2009;16(5):860–9. 19436224 10.1097/gme.0b013e3181a3cdaf PMC2743857

[11] Williams RE, Levine KB, Kalilani L, Lewis J, Clark RV. Menopause-specific questionnaire assessment in US population-based study shows negative impact on health-related quality of life. Maturitas. 2009;62(2):153–9. 19157732 10.1016/j.maturitas.2008.12.006

[12] Daly E, Gray A, Barlow D, McPherson K, Roche M, Vessey M. Measuring the impact of menopausal symptoms on quality of life. BMJ. 1993;307(6908):836–40. 8401125 10.1136/bmj.307.6908.836 PMC1678884

[13] Soares CN. Depression and menopause. Med Clin North Am. 2019;103(4):651–67. 31078198 10.1016/j.mcna.2019.03.001

[14] Bromberger JT, Kravitz HM, Chang YF, Cyranowski JM, Brown C, Matthews KA. Major depression during and after the menopausal transition: study of women’s health across the Nation (SWAN). Psychol Med. 2011;41(09):1879–88. 21306662 10.1017/S003329171100016X PMC3584692

[15] Hart J, Menopause. Shifting hormones linked to anxiety and depression symptoms. Altern Complement Ther. 2019;25(5):254–6.

[16] Usall J, Pinto-Meza A, Fernández A, de Graaf R, Demyttenaere K, Alonso J, et al. Suicide ideation across reproductive life cycle of women results from a European epidemiological study. J Affect Disord. 2009;116(1–2):144–7. 19155069 10.1016/j.jad.2008.12.006

[17] Caretto M, Simoncini T. Hormone replacement therapy (HRT). Endocrinology. 2020;1–18.

[18] Schmidt PJ, Nieman L, Danaceau MA, Tobin MB, Roca CA, Murphy JH, et al. Estrogen replacement in perimenopause-related depression: A preliminary report. Am J Obstet Gynecol. 2000;183(2):414–20. 10942479 10.1067/mob.2000.106004

[19] Soares C, Almeida OP, Joffe H, Cohen LS. Efficacy of estradiol for the treatment of depressive disorders in perimenopausal women. Arch Gen Psychiatry. 2001;58(6):529. 11386980 10.1001/archpsyc.58.6.529

[20] Schmidt PJ, Ben Dor R, Martinez PE, Guerrieri GM, Harsh VL, Thompson K, et al. Effects of estradiol withdrawal on mood in women with past perimenopausal depression. JAMA Psychiatry. 2015;72(7):714. 26018333 10.1001/jamapsychiatry.2015.0111 PMC6391160

[21] Yang JL, Hodara E, Sriprasert Intira, Shoupe D, Stanczyk FZ. Estrogen deficiency in the menopause and the role of hormone therapy: integrating the findings of basic science research with clinical trials. Menopause. 2024;31(10):926–39. 39081162 10.1097/GME.0000000000002407 PMC12072814

[22] NICE, Menopause. identification and management [Internet]. NICE; 2024 [updated 2024; cited 2025 Jun 25]. Available from: https://www.nice.org.uk/guidance/ng23/chapter/recommendations

[23] Graziottin A, Serafini A. Depression and the menopause: why antidepressants are not enough? Menopause Int. 2009;15(2):76–81. 19465674 10.1258/mi.2009.009021

[24] Glynne S, Newson L. Curb antidepressant use: perimenopausal women May benefit from HRT. BMJ. 2024;384:q220. 38286465 10.1136/bmj.q220

[25] Martin-Key NA, Funnell EL, Spadaro B, Bahn S. Perceptions of healthcare provision throughout the menopause in the UK: a mixed-methods study. Npj Women’s Health. 2023;1(1):1–10.

[26] National Institute for Health and Care Excellence. Clinical knowledge summary. Hormone replacement therapy (HRT). 2022. Available from: https://cks.nice.org.uk/topics/menopause/prescribing-information/hormone-replacement-therapy-hrt/ [Accessed 3 April 2024].

[27] British Menopause Society. Cognitive Behaviour Therapy (CBT) for Menopausal Symptoms. 2019. Available from: https://thebms.org.uk/wp-content/uploads/2022/12/01-BMS-TfC-CBT-NOV2022-A.pdf [Accessed 25 June 2025].

[28] Lumsden MA, Davies M, Sarri G, Guideline Development Group for Menopause. Diagnosis and management (NICE clinical guideline 23). Diagnosis and management of menopause: the National Institute of health and care excellence (NICE) guideline. JAMA Intern Med. 2016;176(8):1205–6. 27322881 10.1001/jamainternmed.2016.2761

[29] Scott A, Newson L. Should we be prescribing testosterone to perimenopausal and menopausal women? A guide to prescribing testosterone for women in primary care. Br J Gen Pract. 2020;70(693):203–4. 32217602 10.3399/bjgp20X709265 PMC7098532

[30] Islam RM, Bell RJ, Green S, Page MJ, Davis SR. Safety and efficacy of testosterone for women: a systematic review and meta-analysis of randomised controlled trial data. Lancet Diabetes Endocrinol. 2019;7(10). 10.1016/S2213-8587(19)30189-5 31353194

[31] Hamoda H. Availability of menopausal hormone therapy products worldwide. Maturitas. 2020;141:87–8. 32061438 10.1016/j.maturitas.2020.02.002

[32] Martin-Key NA, Funnell EL, Benacek J, Spadaro B, Bahn S. Using network analysis to understand the association between menopause and depressive symptoms. Npj Womens Health. 2024;2:41.

[33] Martin-Key NA, Funnell EL, Benacek J, Spadaro B, Bahn S. Intention to use a mental health app for menopause: health belief model approach. JMIR Form Res. 2024;8:e60434. 39412868 10.2196/60434 PMC11525080

[34] Martin-Key NA, Funnell EL, Spadaro B, Bahn S. Perceptions of healthcare provision throughout the menopause in the UK: a mixed-methods study. Npj Womens Health. 2023;1:2.

[35] El Khoudary SR, Greendale G, Crawford SL, Avis NE, Brooks MM, Thurston RC, et al. The menopause transition and womenʼs health at midlife. Menopause. 2019;26(10):1213–27. 31568098 10.1097/GME.0000000000001424 PMC6784846

[36] Hilditch JR, Lewis J, Peter A, van Maris B, Ross A, Franssen E, et al. A menopause-specific quality of life questionnaire: development and psychometric properties. Maturitas. 1996;24(3):161–75. 8844630 10.1016/s0378-5122(96)82006-8

[37] Sijtsma K, Ellis JL, Borsboom D. Recognize the value of the sum score, psychometrics’ greatest accomplishment. Psychometrika. 2024;89(1):84–117. 38627311 10.1007/s11336-024-09964-7 PMC11588849

[38] Widaman KF, Revelle W. Thinking thrice about sum scores, and then some more about measurement and analysis. Behav Res Methods. 2023;55(2):788–806. 35469086 10.3758/s13428-022-01849-w PMC10027776

[39] Schultz NM, Morga A, Siddiqui E, Rhoten SE. Psychometric evaluation of the MENQOL instrument in women experiencing vasomotor symptoms associated with menopause. Adv Ther. 2024;41(6):2233–52. 38396203 10.1007/s12325-024-02787-z PMC11133125

[40] Lewis JE, Hilditch JR, Wong CJ. Further psychometric property development of the Menopause-Specific quality of life questionnaire and development of a modified version, MENQOL-Intervention questionnaire. Maturitas. 2005;50(3):209–21. 15734602 10.1016/j.maturitas.2004.06.015

[41] Cohen J. Statistical power analysis for the behavioral sciences. 2nd ed. New York: Academic; 1988.

[42] Duffy O, Iversen L, Hannaford PC. The menopause it’s somewhere between a taboo and a joke. A focus group study. Climacteric. 2011;14(4):497–505. 21395452 10.3109/13697137.2010.549974

[43] Hyde A, Nee J, Drennan J, Butler M, Howlett E. Hormone therapy and the medical encounter. Menopause. 2010;17(2):344–50. 20124923 10.1097/gme.0b013e3181c6b26f

[44] Hunter MS, Liao K-M. Evaluation of a four-session cognitive-behavioral intervention for menopausal hot flushes. Br J Health Psychol. 1996;1:113–25.

[45] Stefanopoulou E, Hunter MS. Telephone-guided Self-Help cognitive behavioural therapy for menopausal symptoms. Maturitas. 2014;77(1):73–7. 24144959 10.1016/j.maturitas.2013.09.013

[46] Hardy C, Griffiths A, Norton S, Hunter MS. Self-help cognitive behavior therapy for working women with problematic hot flushes and night sweats (MENOS@Work): a multicenter randomized controlled trial. Menopause. 2018;25(5):508–19. 29315132 10.1097/GME.0000000000001048

[47] Hunter MS, Coventry S, Hamed H, Fentiman I, Grunfeld EA. Evaluation of a group cognitive behavioural intervention for women suffering from menopausal symptoms following breast cancer treatment. Psycho-oncology. 2009;18(5):560–3. 18646246 10.1002/pon.1414

[48] Ayers B, Smith M, Hellier J, Mann E, Hunter MS. Effectiveness of group and self-help cognitive behavior therapy in reducing problematic menopausal hot flushes and night sweats (MENOS 2). Menopause: J North Am Menopause Soc. 2012;19(7):749–59. 10.1097/gme.0b013e31823fe835 22336748

[49] Spector A, Li Z, He L, Badawy Y, Desai R. The effectiveness of psychosocial interventions on non-physiological symptoms of menopause: A systematic review and meta-analysis. J Affect Disord. 2024;352:460–72. 38364979 10.1016/j.jad.2024.02.048

[50] Balabanovic J, Ayers B, Hunter MS. Cognitive behaviour therapy for menopausal hot flushes and night sweats: A qualitative analysis of women’s experiences of group and Self-Help CBT. Behav Cogn Psychother. 2012;41(4):441–57. 22947108 10.1017/S1352465812000677

[51] Knowles SE, Toms G, Sanders C, Bee P, Lovell K, Rennick-Egglestone S et al. Qualitative Meta-Synthesis of User Experience of Computerised Therapy for Depression and Anxiety. Harris F, editor. PLoS ONE. 2014;9(1):e84323. 10.1371/journal.pone.0084323 PMC3894944 24465404

[52] Kim JH, Yu HJ. The effectiveness of cognitive behavioral therapy on depression and sleep problems for climacteric women: A systematic review and Meta-Analysis. J Clin Med. 2024;13(2):412. 38256545 10.3390/jcm13020412 PMC10816049

[53] Martin-Key NA, Spadaro B, Schei TS, Bahn S. Proof-of-Concept support for the development and implementation of a digital assessment for perinatal mental health: mixed methods study. J Med Internet Res. 2021;23(6):e27132. 34033582 10.2196/27132 PMC8183599

[54] Smith S, Paparo J, Wootton BM. Understanding psychological treatment barriers, preferences and histories of individuals with clinically significant depressive symptoms in australia: a preliminary study. Clin Psychol. 2021;25(2):223–33.

[55] Davis SR, Baber R, Panay N, Bitzer J, Perez SC, Islam RM, Kaunitz AM, Kingsberg SA, Lambrinoudaki I, Liu J, Parish SJ, Pinkerton J, Rymer J, Simon JA, Vignozzi L, Wierman ME. Global consensus position statement on the use of testosterone therapy for women. J Clin Endocrinol Metabolism. 2019;104(10):4660–6. 10.1210/jc.2019-01603 PMC6821450 31498871

[56] Davis SR, Wahlin-Jacobsen S. Testosterone in women–the clinical significance. Lancet Diabetes Endocrinol. 2015;3(12):980–92. 26358173 10.1016/S2213-8587(15)00284-3

[57] Macpherson BE, Quinton ND. Menopause and healthcare professional education: A scoping review. Maturitas. 2022;166:89–95. 36095904 10.1016/j.maturitas.2022.08.009

[58] Barber K, Charles A. Barriers to accessing effective treatment and support for menopausal symptoms: A qualitative study capturing the behaviours, beliefs and experiences of key stakeholders. Patient Prefer Adherence. 2023;17:2971–80. 38027078 10.2147/PPA.S430203 PMC10657761

[59] Farah D, Ceccaldi PF, Farah L, Ayoubi JM, Vallée A. Willingness to use clinical scales for menopause management among general practitioners. Climacteric. 2024;27(6):555–60. 39254442 10.1080/13697137.2024.2395986

[60] Kinnersley P, Edwards A, Hood K, Ryan R, Prout H, Cadbury N, et al. Interventions before consultations to help patients address their information needs by encouraging question asking: systematic review. BMJ. 2008;337(jul16 1):a485–5. 18632672 10.1136/bmj.a485 PMC2500196

[61] Turner GM, Mullis R, Lim L, Kreit L, Mant J. Using a checklist to facilitate management of long-term care needs after stroke: insights from focus groups and a feasibility study. BMC Fam Pract. 2019;20(1). 10.1186/s12875-018-0894-3 PMC6318919 30609920

[62] Matson TE, Hallgren KA, Lapham GT, Oliver M, Wang X, Williams EC, et al. Psychometric performance of a substance use symptom checklist to help clinicians assess substance use disorder in primary care. JAMA Netw Open. 2023;6(5):e2316283–3. 37234003 10.1001/jamanetworkopen.2023.16283 PMC10220521

[63] Pershad A, Morris JM, Pace D, Khanna P. Racial disparities in menopausal hormone therapy acceptance: a pilot study. Menopause. 2022;29(11):1263–8. 36067406 10.1097/GME.0000000000002061

