# Supplementary material for: Differential symptom relief profiles of menopausal therapies: an online survey study
Source: BMC Womens Health. 2025 Aug 4;25:384. doi: 10.1186/s12905-025-03929-3 (PMC12323212; doi:10.1186/s12905-025-03929-3)
Supplement: Supplementary file 1 — Supplementary Material 1 [file 12905_2025_3929_MOESM1_ESM.docx]

**Supplementary Information**

**
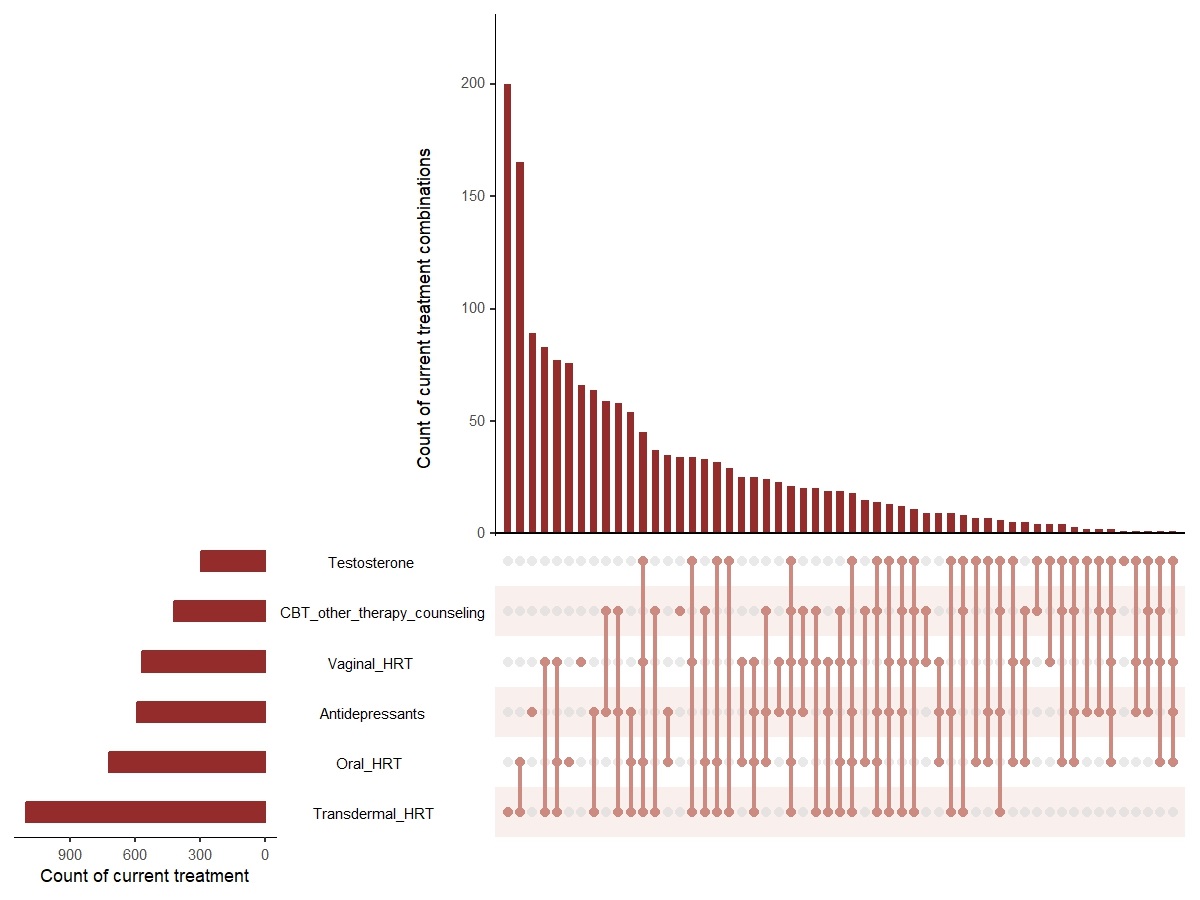
**

**Supplementary Figure 1.** Frequency of current treatment combinations (any length of use) and overall frequency of current treatment use (any length of use) per treatment option. ***Key.*** CBT, cognitive behavioral therapy; HRT, hormone replacement therapy.

| **Supplementary Table 1.** Treatment response (% responders) for the 29 menopausal symptoms from the Menopause-Specific Quality of Life Questionnaire (MENQOL) per treatment (current use ≥3months) | | | | | | |
| --- | --- | --- | --- | --- | --- | --- |
|  | **Transdermal HRT**  **(*n*=871)** | **Oral HRT**  **(*n*=470)** | **Vaginal HRT**  **(*n*=347)** | **Antidepressants**  **(*n*=356)** | **Testosterone**  **(*n*=180)** | **CBT/other therapy/**  **counselling**  **(*n*=146 ^a^)** |
|  | ***n* (%)** | | | | | |
| Hot flushes | 527 (60.51) | 235 (50.00) | 22 (6.34) | 30 (8.43) | 11 (6.11) | 1 (0.68) |
| Night sweats | 506 (58.09) | 232 (49.36) | 18 (5.19) | 18 (5.06) | 16 (8.89) | 1 (0.68) |
| Sweating | 190 (21.81) | 98 (20.85) | 10 (2.88) | 10 (2.81) | 7 (3.89) | 1 (0.68) |
| Being dissatisfied with my personal life | 163 (18.71) | 48 (10.21) | 10 (2.88) | 79 (63.20) | 17 (9.44) | 77 (52.74) |
| Feeling anxious/nervous | 361 (41.45) | 140 (29.79) | 12 (3.46) | 225 (63.20) | 29 (16.11) | 112 (76.71) |
| Experiencing poor memory | 280 (32.15) | 86 (18.30) | 9 (2.59) | 23 (6.46) | 50 (27.78) | 6 (4.11) |
| Accomplishing less than I used to | 161 (18.48) | 46 (9.79) | 4 (1.15) | 24 (6.74) | 43 (23.89) | 33 (22.60) |
| Feeling depressed/down/blue | 258 (29.62) | 75 (15.96) | 6 (1.73) | 181 (50.84) | 36 (20.00) | 81 (55.48) |
| Being impatient with other people | 302 (34.67) | 100 (21.28) | 6 (1.73) | 88 (24.72) | 21 (11.67) | 43 (29.45) |
| Feelings of wanting to be alone | 102 (11.71) | 29 (6.17) | 3 (0.86) | 53 (14.89) | 16 (8.89) | 33 (22.60) |
| Flatulence/gas pains | 35 (4.02) | 14 (2.98) | 2 (0.58) | 3 (0.84) | 0 (0.00) | 1 (0.68) |
| Aching in muscles/joints | 303 (34.79) | 88 (18.72) | 9 (2.59) | 15 (4.21) | 41 (22.78) | 2 (1.37) |
| Feeling tired/worn out | 239 (27.44) | 80 (17.02) | 6 (1.73) | 30 (8.43) | 70 (38.89) | 11 (7.53) |
| Difficulty sleeping | 382 (43.86) | 226 (48.09) | 12 (3.46) | 63 (17.70) | 19 (10.56) | 19 (13.01) |
| Aches in back of neck/head | 66 (7.58) | 25 (5.32) | 1 (0.29) | 4 (1.12) | 4 (2.22) | 3 (2.05) |
| Decrease in physical strength | 68 (7.81) | 21 (4.47) | 3 (0.86) | 3 (0.84) | 69 (38.33) | 2 (1.37) |
| Decrease in stamina | 80 (9.18) | 25 (5.32) | 3 (0.86) | 4 (1.12) | 72 (40.00) | 3 (2.05) |
| Feeling a lack of energy | 185 (21.24) | 72 (15.32) | 4 (1.15) | 29 (8.15) | 97 (53.89) | 12 (8.22) |
| Dry skin | 105 (12.06) | 33 (7.02) | 8 (2.31) | 4 (1.12) | 5 (2.78) | 0 (0.00) |
| Weight gain | 79 (9.07) | 45 (9.57) | 5 (1.44) | 7 (1.97) | 14 (7.78) | 1 (0.68) |
| Increased facial hair | 29 (3.33) | 10 (2.13) | 2 (0.58) | 2 (0.56) | 1 (0.56) | 1 (0.68) |
| Changes in appearance/texture/tone of skin | 58 (6.66) | 18 (3.83) | 6 (1.73) | 2 (0.56) | 7 (3.89) | 1 (0.68) |
| Feeling bloated | 63 (7.23) | 27 (5.74) | 2 (0.58) | 2 (0.56) | 1 (0.56) | 1 (0.68) |
| Lower backache | 46 (5.28) | 20 (4.26) | 2 (0.58) | 4 (1.12) | 5 (2.78) | 1 (0.68) |
| Frequent urination | 106 (12.17) | 32 (6.81) | 98 (28.24) | 3 (0.84) | 3 (1.67) | 1 (0.68) |
| Involuntary urination when laughing/coughing | 54 (6.20) | 14 (2.98) | 47 (13.54) | 2 (0.56) | 1 (0.56) | 0 (0.00) |
| Change in sexual desire | 119 (13.66) | 42 (8.94) | 45 (12.97) | 6 (1.69) | 102 (56.67) | 3 (2.05) |
| Vaginal dryness during intercourse | 161 (18.48) | 54 (11.49) | 244 (70.32) | 3 (0.84) | 9 (5.00) | 1 (0.68) |
| **eTable 1.** Treatment response (% responders) for the 29 menopausal symptoms from the Menopause-Specific Quality of Life Questionnaire (MENQOL) per treatment (current use ≥3months) | | | | | | |
| Avoiding intimacy | 61 (7.00) | 18 (3.83) | 47 (13.54) | 5 (1.40) | 42 (23.33) | 9 (6.16) |

***Note.*** CBT, cognitive behavioral therapy; HCP, health care professional; HRT, hormone replacement therapy

***Key.*** ^a^ Missing data from one respondent
